# Results of a 10‐year survey of workload for 10 treatment vaults at a high‐throughput comprehensive cancer center

**DOI:** 10.1002/acm2.12076

**Published:** 2017-04-19

**Authors:** Ziad H. Saleh, Jeho Jeong, Brian Quinn, James Mechalakos, Jean St. Germain, Lawrence T. Dauer

**Affiliations:** ^1^ Department of Medical Physics Memorial Sloan Kettering Cancer Center York Ave NY USA; ^2^ Department of Radiology Memorial Sloan Kettering Cancer Center York Ave NY USA

**Keywords:** Linear Accelerators (Linacs), modulation factor, radiation safety, shielding design, workload

## Abstract

The workload for shielding purposes of modern linear accelerators (linacs) consists of primary and scatter radiation which depends on the dose delivered to isocenter (cGy) and leakage radiation which depends on the monitor units (MUs). In this study, we report on the workload for 10 treatment vaults in terms of dose to isocenter (cGy), monitor units delivered (MUs), number of treatment sessions (Txs), as well as, use factors (U) and modulation factors (CI) for different treatment techniques. The survey was performed for the years between 2006 and 2015 and included 16 treatment machines which represent different generations of Varian linear accelerators (6EX, 600C, 2100C, 2100EX, and TrueBeam) operating at different electron and x‐ray energies (6, 9, 12, 16 and 20 MeV electrons and, 6 and 15 MV x‐rays). An institutional review board (IRB) approval was acquired to perform this study. Data regarding patient workload, dose to isocenter, number of monitor units delivered, beam energies, gantry angles, and treatment techniques were exported from an ARIA treatment management system (Varian Medical Systems, Palo Alto, Ca.) into Excel spreadsheets and data analysis was performed in Matlab. The average (± std‐dev) number of treatment sessions, dose to isocenter, and number of monitor units delivered per week per machine in 2006 was 119 ± 39 Txs, (300 ± 116) × 10^2^ cGys, and (78 ± 28) × 10^3^ MUs respectively. In contrast, the workload in 2015 was 112 ± 40 Txs, (337 ± 124) × 10^2^ cGys, and (111 ± 46) × 10^3^ MUs. 60% of the workload (cGy) was delivered using 6 MV and 30% using 15 MV while the remaining 10% was delivered using electron beams. The modulation factors (MU/cGy) for IMRT and VMAT were 5.0 (± 3.4) and 4.6 (± 1.6) respectively. Use factors using 90**°** gantry angle intervals were equally distributed (~0.25) but varied considerably among different treatment techniques. The workload, in terms of dose to isocenter (cGy) and subsequently monitor units (MUs), has been steadily increasing over the past decade. This increase can be attributed to increased use of high dose hypo‐fractionated regimens (SBRT, SRS) and the increase in use of IMRT and VMAT, which require higher MUs per cGy as compared to more conventional treatment (3DCRT). Meanwhile, the patient workload in terms of treatment sessions per week remained relatively constant. The findings of this report show that variables used for shielding purposes still fall within the recommendation of NCRP Report 151.

## 1 Introduction

The National Council on Radiation Protection and Measurements (NCRP) has offered helpful recommendations and technical information related to the design and installation of structural shielding for megavoltage x‐ray radiotherapy facilities in Report 151.^1^ For primary barrier shielding considerations due to primary and scatter radiation, the most important parameter is the workload, defined as the time integral of the absorbed‐dose rate determined as the depth of maximum absorbed dose, at 1 m from the source.^1^ When designing and evaluating linac shielding, the value for workload is usually specified as the absorbed dose delivered in cGy to the isocenter in a week, and is selected for each accelerator based on its projected use. This is usually estimated from the number of patients (or fields) treated in a week and the absorbed dose delivered per patient (or field).^1^

Shielding calculation for secondary barrier, due to leakage radiation in the treatment head, depends largely on the monitor units delivered (MU).^2^, ^3^ Intensity modulated radiation therapy (IMRT) has become the standard of care in the treatment of prostate, head and neck (H&N) and other sites.^4^, ^5^ IMRT delivery is inefficient due to multi‐leaf collimator (MLC) modulation and requires more MU per treatment.^4^ Previous reports have shown that machines treating with IMRT have higher workloads than non‐IMRT machines^3^, ^6^, ^7^ and that workloads for IMRT machines could reach approximately 100,000 MU/wk. The NCRP recognizes this difference in workload for IMRT treatments relative to three‐dimensional conformal radiotherapy (3DCRT), considers the increase in leakage associated with the higher workloads when designing shielding for an IMRT room, and utilizes a modulation factor, which they term the “IMRT factor” (CI), to include the increased leakage workload in secondary barrier analyses.^1^ Values of CI can range from 2 to 10 or more.^3^, ^7^, ^8^, ^9^ In addition, the increased leakage due to higher MU for high energy x‐ray beams (>10 MV) can lead to an increase in production of neutrons.^1^, ^10^

Since the publication of these earlier reports, volumetric modulated arc therapy (VMAT) technique was introduced into the clinic. VMAT allows the simultaneous modulation of MLC, gantry speed, dose rate, and faster delivery time.^11^, ^12^, ^13^, ^14^ Similar to IMRT, VMAT requires higher MU per treatment compared to conventional 3DCRT.^15^ The increase in monitor units for IMRT and VMAT does not appreciably affect the amount of radiation reaching the primary barrier on a per‐plan basis, since the prescription dose, and consequently, the amount of radiation reaching the primary barrier stays the same.^1^, ^7^ If, however, more patients are treated per day, due to lower overall setup and treatment time, the total weekly dose to the primary barrier will increase.

Image guided radiotherapy treatments (IGRT) has also become very common due to the availability of onboard kV imagers (kV‐OBI) and MV electronic portal imager devices (EPID) on modern linacs which allows accurate patient positioning and tumor monitoring during delivery.^16^ IGRT is widely used in the delivery of hypo‐fractionated regimens such as stereotactic body radiotherapy (SBRT) and single fraction cranial stereotactic radiosurgery (SRS) which use higher dose per fraction.^17^ However, the increase in setup and treatment time can result in lower patient throughput.

Therefore, it would be instructive to examine the impact on monitor units associated with the various modern clinical treatment techniques, as well as the overall impact on patient load, and treatment energy distribution. In this study, we wish to obtain a clearer picture of the contribution of current treatment techniques to machine workload, and to evaluate patient loads. In addition, we evaluate the use factor (U), defined as the fraction of a primary‐beam workload that is directed toward a given primary barrier, which will depend significantly on the type of radiation installation and modality. In order to significantly expand on earlier studies we extracted 10 years worth of data on 16 treatment machines.

## 2 Materials and methods

To perform this survey, we utilized data from 16 Varian Linear Accelerators (LINACs) that were operational at different time periods between 2006 and 2015 in 10 treatment vaults at the main center in our institution as illustrated in Table 1. The linacs represent different generations of Varian machines (6EX, 600C, 2100C, 2100EX, and TrueBeam) operating at different electron and photon energies (6, 9, 12, 16 and 20 MeV electrons and, 6 and 15 MV photons). We note that some machines in certain rooms were de‐commissioned during this 10 yr period and were replaced primarily with state‐of‐the‐art TrueBeam machines with one exception where the vault (#6) could not accept the TrueBeam accelerator and a Varian 6EX was installed (Table 1).

**Table 1:** Summary of treatment machines, beam energies, techniques, and data availability

Vault #	Model	Photons	Electrons	Treatment techniques	Data availability
		[MV]	[MeV]		
1	2100EX	6, 15	6, 9, 12, 16, 20	3DCRT, IMRT	01/01/2006–12/31/2015
2	6EX	6	–	3DCRT, IMRT	07/11/2006–12/31/2015
3	6EX	6	–	3DCRT, IMRT	01/01/2006–12/31/2015
4	2100EX	6, 15	6, 9, 12, 16, 20	3DCRT, IMRT	01/01/2006–06/20/2014
	True Beam	6, 15	6, 9, 12, 16, 20	3DCRT, IMRT, VMAT, SBRT, TBI	04/20/2015–12/31/2015
5	2100C	6, 15	6, 9, 12, 16, 20	3DCRT, IMRT, TBI	01/01/2006–06/14/2011
	True Beam	6, 15	6, 9, 12, 16, 20	3DCRT, IMRT, VMAT, SBRT	02/28/2013–12/31/2015
6	600C	6	–	3DCRT, IMRT	01/01/2006–05/17/2013
	6EX	6	–	3DCRT, IMRT	03/17/2014–12/31/2015
7	600C	6	–	3DCRT, IMRT	01/01/2006–05/20/2010
	True Beam	6, 15	6, 9, 12, 16, 20	3DCRT, IMRT, VMAT, SBRT	05/02/2011–12/31/2015
8	600C	6	–	3DCRT	01/01/2006–07/26/2006
	2300IX1	6, 15	6, 9, 12, 16, 20	3DCRT, IMRT, SBRT	07/20/2007–12/31/2015
9	2100EX	6, 15	6, 9, 12, 16, 20	3DCRT, IMRT, TBI, TSEB	01/01/2006–12/31/2015
10	2100C	6, 15	6, 9, 12, 16, 20	3DCRT, IMRT, TSEB	01/01/2006–10/12/2007
	True Beam	6, 15	6, 9, 12, 16, 20	3DCRT, IMRT, VMAT, SBRT, TSEB	03/30/2010–12/31/2015

At our institution, some machines are designated for specific treatment sites based on machine energy and the availability of ancillary devices (ExacTrac, Calypso, Align RT, 6DOF couch, etc.). As an example, the machine in vault #4 is used primarily for total body irradiation (TBI) while the machine in vault #9 serves as a backup and the machine in vault #6 is a single energy machine (6 MV) used primarily for treating breast patients and palliative cases.

The treatment parameters in terms of beam energy, gantry angle, Monitor Units (MUs), prescription dose at isocenter (cGy), number of fractions were stored electronically via the ARIA treatment management system (Varian Medical Systems, Palo Alto, Ca.). We note that the transition from an in‐house to a commercial record and verify system (R&V) happened around 1999. The R&V system was upgraded by end of 2005 to Varis 7.0 which resembled the current ARIA interface. The R&V system was followed by several ARIA upgrades (Ver 8.0, 9.0, 10.0, 11.0, 13.6) over the past 10 yr which were backward compatible. We would like to mention that because of machine compatibility issues with R&V system prior to 2005 causing some missing information for some machines, we opted to report on the workload in the past 10 yr.

An IRB approval was acquired to perform this study. Patient treatment records were exported from ARIA using custom reports and stored in Excel spreadsheets. Data were processed in MATLAB (R2011a, Ver 7.12) and anonymized to mask all protected health information (PHI) for further analysis.

All linacs were calibrated per TG‐51 to deliver 1 cGy/MU at 100 SAD for x‐ray beams and 1 cGy/MU at D_max_ (100 SSD) for electron beams using 600 MU/MIN dose rate 18 The workload per machine was calculated based on the number of treatment sessions per week, dose in cGys delivered to isocenter, and the corresponding MUs. An inverse square correction was applied to calculate the dose at isocenter for TBI treatments.

For use factor (U) calculations, the beam angles were binned into 12 bins with 30° gantry angle intervals, and the dose (cGy) delivered by beams in each bin was divided by the total dose (cGy). To estimate use factor for VMAT, knowing the starting and stopping angle along with directionality, each arc was subdivided into 30° intervals. It was assumed that the dose delivered per arc is divided equally among subarcs. For the purpose of this analysis, the gantry angle (0°) is defined at the vertical position (beam down). The modulation factor (CI), for each treatment technique (3DCRT, IMRT, VMAT, SRS, etc.), was computed by dividing the total MU by the total cGy delivered per fraction which resulted in a distribution of CIs.

## 3 Results

**Figure 1:**
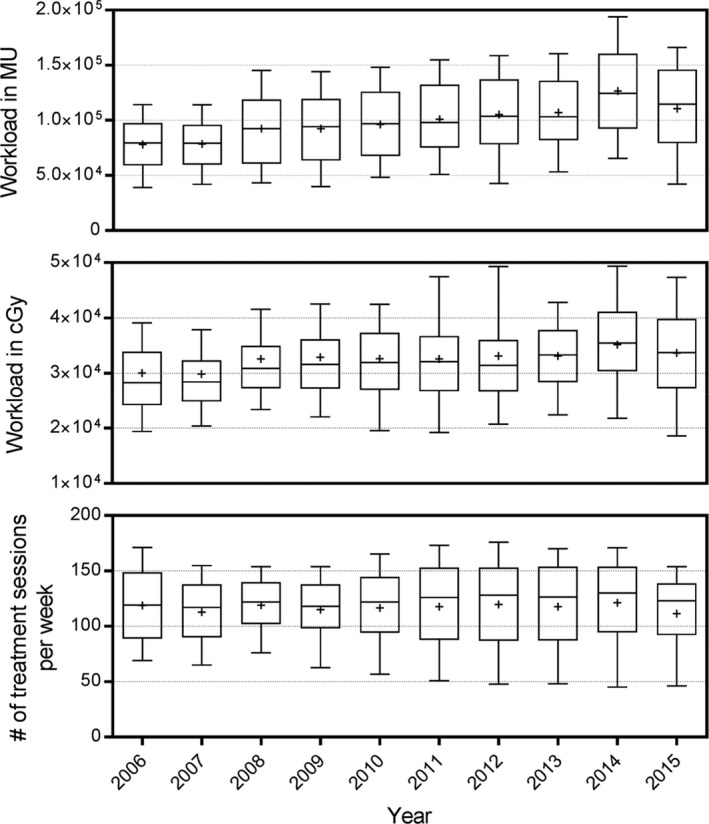
Bar plot representing the workload per machine per week in terms of MU (top panel), dose in cGy (middle panel), and number of treatment sessions (bottom panel) for all machines in all 10 vaults from 2006 to 2015. The bar represents the 25 and 75 percentile and the middle line represents the median while the “+” sign represents the mean. The error bars correspond to one standard deviation.

The workload per week per machine from 2006 to 2015 in terms of Txs, cGy, and MU is shown in Fig. 1. The average (± std‐dev) of Txs, cGys, and MU in 2006 was 119 ± 39 Txs, (300 ± 116) × 10^2^ cGys, and (78 ± 28) × 10^3^ MUs respectively. Meanwhile, the average workload in 2015 was 112 ± 40 Txs, (337 ± 124) × 10^2^ cGys, and (111 ± 46) × 10^3^ MUs. A detailed workload in terms of cGy and MU on a year by year is given in Supplementary Tables S1 and S2.

**Figure 2:**
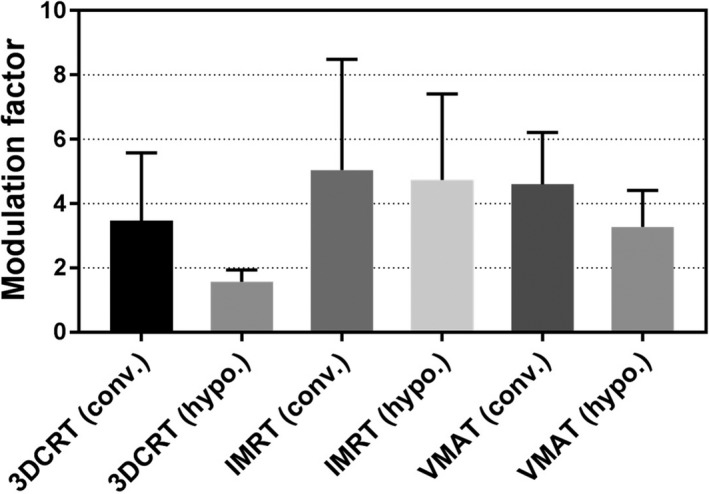
Modulation factor (C_I_) for different treatment delivery techniques on all machines for year 2015. The error bar represents one standard deviation.

**Figure 3:**
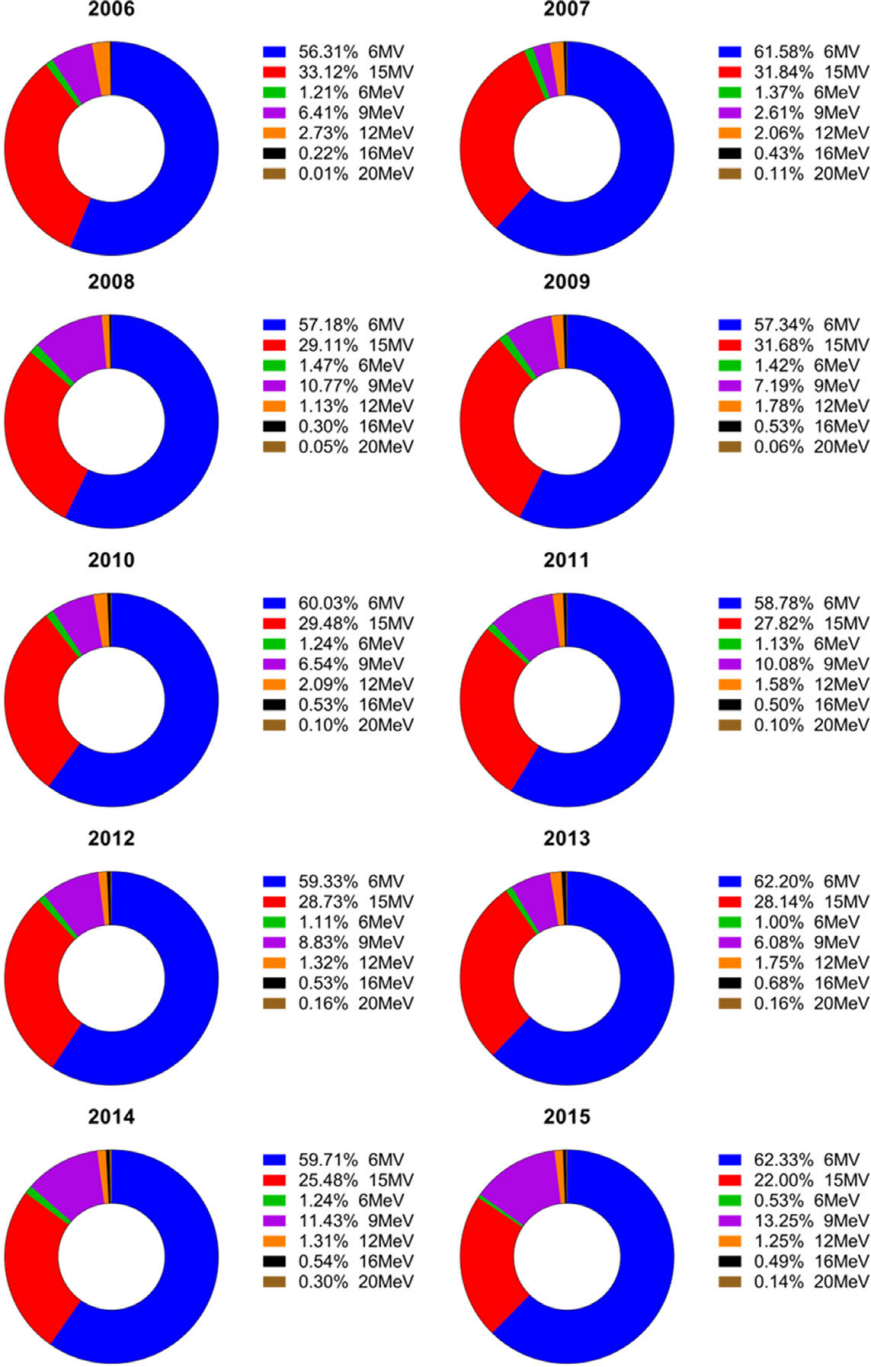
Percentage of workload (cGy) delivered using different x‐ray and electron beam energies for combined treatment techniques on all 10 machines between 2006 and 2015.

The average (± std‐dev) modulation factor (C_I_) was 5.0 (± 3.4) for IMRT, and 4.6 (± 1.6) for VMAT technique as shown in Fig. 2. The average monitor units and dose per fraction for 3DCRT was 398 MU and 295 cGY while for IMRT it was 983 MU and 213 cGY. The fraction of workload (cGy) delivered using 6 MV and 15 MV was 60% and 30%, respectively, while 10% was delivered using electrons as shown in Fig. 3. The percentage of treatments delivered with 3DCRT, IMRT, and VAMT utilizing 6 MV and 15 MV is shown in supplementary Fig. S1.

**Figure 4:**
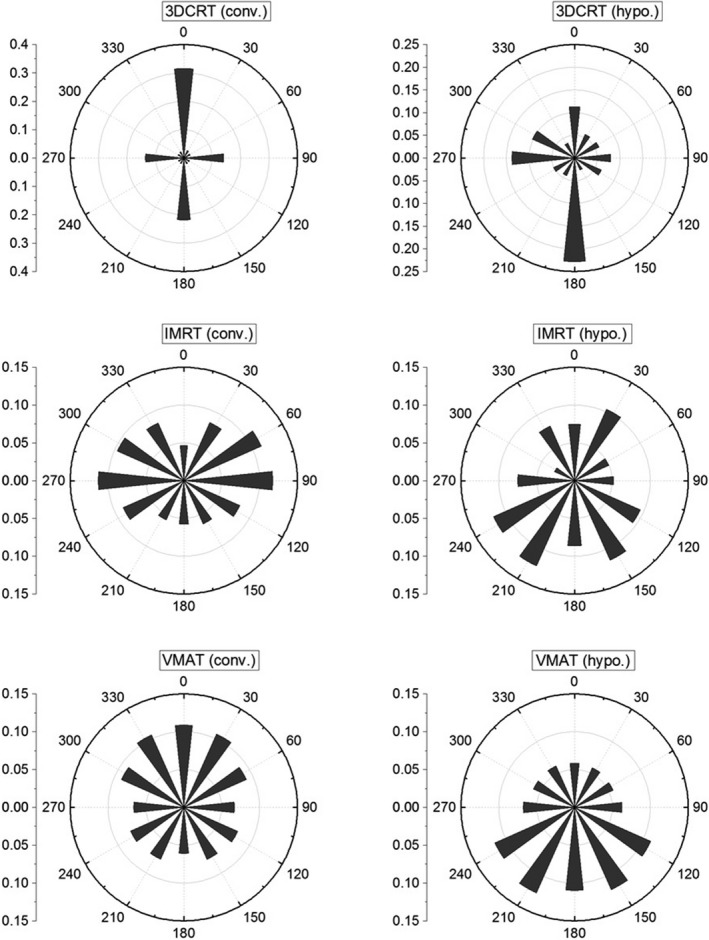
Use factor (U) for different treatment techniques shown in rose plot for all machines in 2015. A total of 12 bins were used with 30° each.

Figure 4 shows the distribution of use factors (U) per treatment technique on all machines in 2015 using 30° gantry‐angle intervals. The use factor in 2015 for each vault (using 90° intervals) is shown in Table S3. The use factor at 270° angle (right) in vault#4 is 0.45 due to TBI treatments and the use factors in vault#6 at 90° and 270° angles were equal to 0.39 due to breast treatment with tangents in supine and prone positions. The use factor of combined treatment techniques in each vault is shown in Fig. S2.

## 4 Discussion

This study constitutes one of the largest surveys of workload at a high throughput comprehensive cancer center. The results of this survey show that the workload in term of cGy and MU has increased by 12% and 42%, respectively, from 2006 to 2015 while the number of treatments per week remained relatively unchanged as shown in Fig. 1. The increase in dose (cGy) can be attributed to increased use of high dose hypo‐fractionated (SBRT) and single fraction (SRS) treatments which consequently lead to increase in MU. Moreover, the additional increase in MU can be attributed to the rapid adoption of IMRT, and most recently VMAT, as standard of care in the treatment delivery of pelvis/prostate and H&N cancer and other sites which require more modulation (MUs per cGy) as compared to more conventional treatment (3DCRT). On the other hand, the use of IGRT for patient positioning and tumor tracking results in longer treatment time and lower throughput.

The increased modulation varied based on the treatment delivery technique as shown in Fig 2. The average modulation factor for IMRT was 5.0 which is slightly higher than that for VMAT (4.7) but similar to previously published results;^3^, ^8^, ^19^ The workload (cGy) delivered using 6 MV (60%) was twice the workload for 15 MV (30%). The 6 MV beam was predominantly used for cranial SRS, lung, and breast treatments while 15 MV beam was used mainly for the treatment pelvis/prostate, and deep seated tumors. The percentage of IMRT and VMAT treatments delivered using 6 MV and 15 MV varied among treatment vaults as shown in Fig. S1.

The use factor was highly dependent on treatment site and delivery technique as shown in Fig 4 and Fig. S2. For example, the use factor was higher toward the ceiling (Up direction) for paraspinal SBRT cases treated with VMAT (hypo). However, these variations become less pronounced when multiple modalities and techniques are combined as shown in Table S3 and the use factor approaches 0.25 with the exception of vault#4 due to TBI treatments and vault#6 due to breast tangents treated in supine and prone positions in addition to opposed beams for palliative treatments.

Based on our survey results, the average patient load in 2015 per machine per week was 112 and the average combined workload (Gy) for 6 MV and 15 MV was 337 Gy which is well below the 500 Gy/Week as recommended by NCRP for dual energy machines. Our results also show that about 33% of the workload is delivered using high energy x‐ray beam (15 MV). IMRT plans treated with 15 MV ranged between 15% and 50% while IMRT plans treated with 6 MV ranged between 30% and 70% depending on the treatment vault. The average workload in MU has increased from 78,000 MU/wk in 2005 to 110,000 MU/wk in 2016. These numbers are slightly higher than those reported by Mechalakos et al. in 2004 due to increased use of IMRT. We also note that some of the machines operated between 8 and 10 hr and therefore these numbers are an overestimate of workload. Our results suggest that NCRP overestimated the workload and it might be adequate to employ Monte Carlo based calculations for shielding design where cost and space might be an issue.

**Figure 5:**
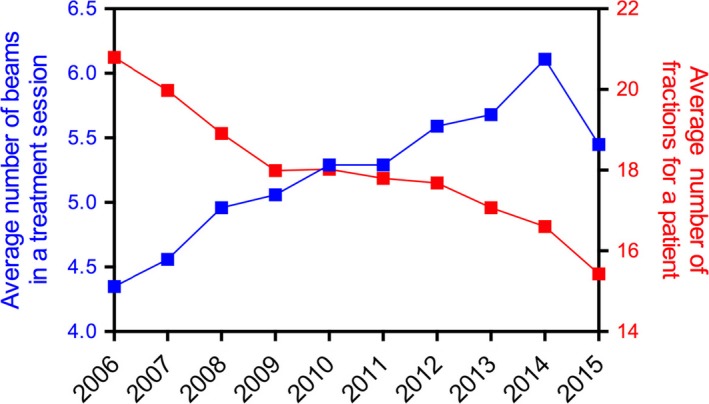
Graph showing the average number of beams per fraction (blue) and number of fractions per patient (red) from 2006 to 2015.

This study also showed that the average number of beams/fraction has been increasing due to the adoption of complex IMRT treatments such as H&N and hypo‐fractionated regimens such as para‐spinal SBRT, and single fraction Cranial SRS which require multiple fields (7–10) compared with the more traditional 3DCRT (4–5 fields) and palliative treatments with two opposed beams (Fig. 5). We note that we upgraded our in‐house treatment planning system (TPS) to commercial TPS in late 2014. The use of VMAT technique in 2015 which utilizes few arcs (2–4) reduced the number of beams slightly. Conversely, the number of fractions per patient has been decreasing with more patients treated with hypo‐fractionated and single fraction. Fig. S3 in the supplementary material illustrate the distribution of use (in cGy) of each treatment technique between 2006 and 2015.

This study did not take into account the extra dose (cGy) and resulting MU from daily and monthly QA routines which are usually done off‐hours. It also ignored the imaging dose generated during patient setup. Moreover, flattening filter free beam (6FFF) has not been heavily utilized at our center but is expected to play a bigger role in SRS treatment because of faster delivery due to its high dose rate.^20^, ^21^ In addition, the use factor for IMRT and VMAT beams can be determined more accurately using EPID dosimetry.

## 5 Conclusion

This study showed that the workload, in terms of dose (cGy) and monitor units (MUs), has been steadily increasing over the past decade. Meanwhile, the patient workload in terms of number of treatment sessions per week remained relatively constant. The findings of this report show that parameters important to shielding evaluations are still within the recommendations of NCRP Report 151 1 even for more modern radiotherapy applications.

## Acknowledgment

The authors thank Michael Sullivan and Anthony Abaya from the Department of Radiation Oncology at Memorial Sloan Kettering Cancer Center for providing the ARIA reports used to extract the data for this analysis. This research was partially supported by the MSK Cancer Center Support Grant/Core Grant (P30 CA008748).

## Conflict of Interest

No conflict of interest.

## Supporting information


**Fig S1**. Percentage of workload (cGy) delivered using different techniques and x‐ray beam energies.
**Fig S2**. Rose plot showing the distribution of use factor (U) at different beam angles for each machine in 2015. 12 bins were used with 30 degrees each.
**Fig S3**. Annual dose (cGy) delivered per technique on all machines for the years between 2006 and 2015. Annual number of patients treated on all machines (right *y*‐axis) has increased slightly.

**Table S1**. Weekly workload per machine in cGy

**Table S2**. Weekly workload per machine in MU

**Table S3**. Use‐factors (U) in 2015 at 90° gantry angle intervals.
