# Supplementary material for: Results of a 10‐year survey of workload for 10 treatment vaults at a high‐throughput comprehensive cancer center
Source: J Appl Clin Med Phys. 2017 Apr 19;18(3):207–14. doi: 10.1002/acm2.12076 (PMC5689849; doi:10.1002/acm2.12076)
Supplement: Supplementary file 1 — Fig S1. Percentage of workload (cGy) delivered using different techniques and x‐ray beam energies. Fig S2. Rose plot showing the distribution of use factor (U) at different beam angles for each machine in 2015. 12 bins were used with 30 degrees each. Fig S3. Annual dose (cGy) delivered per technique on all machines for the years between 2006 and 2015. Annual number of patients treated on all machines (right y‐axis) has increased slightly. [file ACM2-18-207-s001.docx]

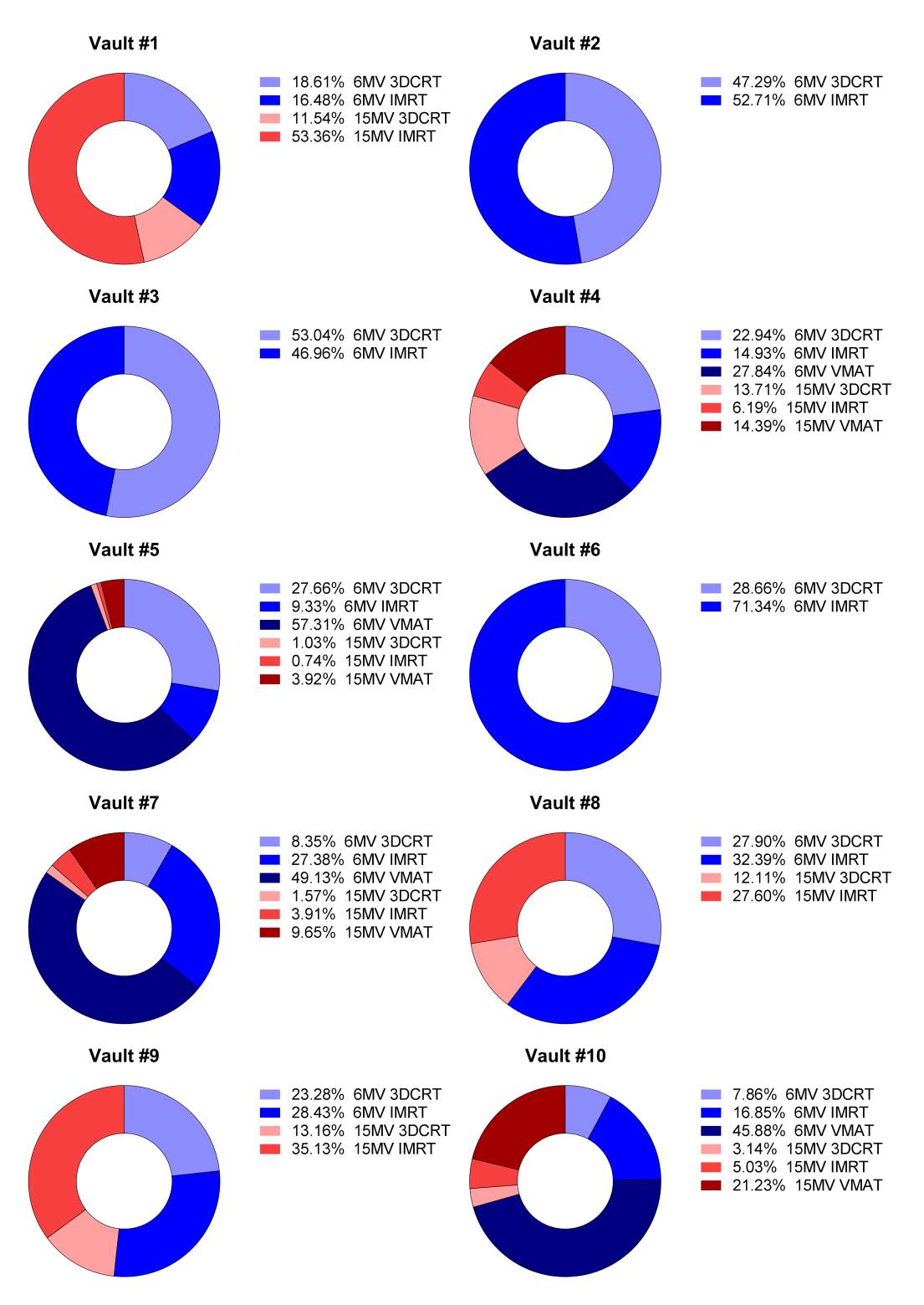


**Fig S1.** Percentage of workload (cGy) delivered using different techniques and x-ray beam energies.





**Fig S2.** Rose plot showing the distribution of use factor (U) at different beam angles for each machine in 2015. 12 bins were used with 30 degrees each.





**Fig S3.** Annual dose (cGy) delivered per technique on all machines for the years between 2006 and 2015. Annual number of patients treated on all machines (right y-axis) has increased slightly.

| **Table S1. Weekly workload per machine in cGy** | | | | | | | | | | |
| --- | --- | --- | --- | --- | --- | --- | --- | --- | --- | --- |
|  | **Year** | | | | | | | | | |
|  | **2006** | **2007** | **2008** | **2009** | **2010** | **2011** | **2012** | **2013** | **2014** | **2015** |
| min | 1050 | 405 | 500 | 3050 | 1440 | 940 | 2985 | 500 | 1500 | 2010 |
| 1st quarter | 24128 | 24801 | 27305 | 27263 | 27011 | 26764 | 26731 | 28434 | 30364 | 27300 |
| median | 28342 | 28478 | 30900 | 31611 | 31961 | 32113 | 31436 | 33328 | 35490 | 33742 |
| 3rd quarter | 33936 | 32382 | 35026 | 36161 | 37353 | 36783 | 36034 | 37833 | 41148 | 39900 |
| max | 117832 | 106154 | 110580 | 106442 | 99062 | 109586 | 106486 | 84042 | 102598 | 84269 |

| **Table S2. Weekly workload per machine in MU** | | | | | | | | | | |
| --- | --- | --- | --- | --- | --- | --- | --- | --- | --- | --- |
|  | **Year** | | | | | | | | | |
|  | **2006** | **2007** | **2008** | **2009** | **2010** | **2011** | **2012** | **2013** | **2014** | **2015** |
| min | 1240 | 925 | 644 | 8250 | 6086 | 2872 | 9780 | 1810 | 2148 | 3807 |
| 1st quarter | 58972 | 59726 | 60678 | 63665 | 67699 | 75188 | 78047 | 81884 | 92373 | 79262 |
| median | 79410 | 79249 | 92594 | 94363 | 96997 | 98034 | 103670 | 103274 | 124464 | 114599 |
| 3rd quarter | 97752 | 96034 | 118920 | 119632 | 126014 | 132414 | 137443 | 136096 | 160696 | 145966 |
| max | 153673 | 153176 | 208037 | 184826 | 176116 | 207011 | 192091 | 226535 | 303243 | 211903 |

| **Table S3: Use-factors (U) in 2015 (90 degree intervals)** | | | | |
| --- | --- | --- | --- | --- |
|  | **0° (down)** | **90° (right)** | **180° (up)** | **270° (left)** |
| **Vault #1** | 28.4% | 23.0% | 26.0% | 22.6% |
| **Vault #2** | 26.1% | 24.7% | 25.1% | 24.1% |
| **Vault #3** | 25.8% | 27.7% | 21.9% | 24.6% |
| **Vault #4** | 24.1% | 12.4% | 18.6% | 45.0% |
| **Vault #5** | 15.0% | 26.8% | 32.7% | 25.4% |
| **Vault #6** | 11.6% | 39.0% | 10.6% | 38.8% |
| **Vault #7** | 33.2% | 20.9% | 24.4% | 21.5% |
| **Vault #8** | 37.4% | 20.2% | 22.7% | 19.7% |
| **Vault #9** | 30.4% | 29.1% | 23.8% | 16.7% |
| **Vault #10** | 32.7% | 21.4% | 23.6% | 22.3% |
